# Application of a Physiologically Based Pharmacokinetic Model to Characterize Time-dependent Metabolism of Voriconazole in Children and Support Dose Optimization

**DOI:** 10.3389/fphar.2021.636097

**Published:** 2021-03-17

**Authors:** Yahui Zhang, Sixuan Zhao, Chuhui Wang, Pengxiang Zhou, Suodi Zhai

**Affiliations:** ^1^ Department of Pharmacy, Peking University Third Hospital, Beijing, China; ^2^ Department of Pharmacy Administration and Clinical Pharmacy, School of Pharmaceutical Sciences, Peking University, Beijing, China

**Keywords:** voriconazole, physiologically based pharmacokinetic model, children, gene, dose optimization

## Abstract

**Background:** Voriconazole is a potent antifungal drug with complex pharmacokinetics caused by time-dependent inhibition and polymorphisms of metabolizing enzymes. It also exhibits different pharmacokinetic characteristics between adults and children. An understanding of these alterations in pharmacokinetics is essential for pediatric dose optimization.

**Objective:** To determine voriconazole plasma exposure in the pediatric population and further investigate optimal dosage regimens.

**Methods:** An adult and pediatric physiologically based pharmacokinetic (PBPK) model of voriconazole, integrating auto-inhibition of cytochrome P450 3A4 (CYP3A4) and *CYP2C19* gene polymorphisms, was developed. The model was evaluated with visual predictive checks and quantitative measures of the predicted/observed ratio of the area under the plasma concentration-time curve (AUC) and maximum concentration (C_max_). The validated pediatric PBPK model was used in simulations to optimize pediatric dosage regimens. The probability of reaching a ratio of free drug (unbound drug concentration) AUC during a 24-h period to minimum inhibitory concentration greater than or equal to 25 (*f*AUC_24h_/MIC ≥ 25) was assessed as the pharmacokinetic/pharmacodynamic index.

**Results:** The developed PBPK model well represented voriconazole's pharmacokinetic characteristics in adults; 78% of predicted/observed AUC ratios and 85% of C_max_ ratios were within the 1.25-fold range. The model maintained satisfactory prediction performance for intravenous administration in pediatric populations after incorporating developmental changes in anatomy/physiology and metabolic enzymes, with all predicted AUC values within 2-fold and 73% of the predicted C_max_ within 1.25-fold of the observed values. The simulation results of the PBPK model suggested that different dosage regimens should be administered to children according to their age, *CYP2C19* genotype, and infectious fungal genera.

**Conclusion:** The PBPK model integrating CYP3A4 auto-inhibition and *CYP2C19* gene polymorphisms successfully predicted voriconazole pharmacokinetics during intravenous administration in children and could further be used to optimize dose strategies. The infectious fungal genera should be considered in clinical settings, and further research with large sample sizes is required to confirm the current findings.

## Introduction

Voriconazole is an essential triazole antifungal agent with *in vivo* activity against a broad spectrum of yeasts and filamentous fungi, commonly used for the prophylaxis and treatment of various invasive fungal infections (IFI) (Clancy and Nguyen, 1998; Saravolatz et al., 2013; Perfect et al., 2003). However, a high interindividual plasma variability has been observed partially due to its nonlinear and time-dependent pharmacokinetics (Purkins et al., 2003; Pfizer, 2010). It also exhibits differences in clearance and bioavailability between adults and children (Schulz et al., 2019). All these factors complicate the successful therapeutic use of voriconazole in pediatric populations.

Voriconazole is a substrate for cytochrome P450 (CYP) enzymes. CYP2C19 is the primary enzyme that contributes to the main circulating metabolite of voriconazole, voriconazole N-oxide, while CYP3A4 and CYP2C9 are also responsible for its metabolism (Hyland et al., 2003). Only 2% of voriconazole is excreted unmetabolized in the urine (Levêque et al., 2006). The time-dependent inhibition (TDI) of CYP3A4 observed in *in vitro* studies (Li et al., 2020) may play a role in the elevated exposure to voriconazole at multiple doses, which could not be predicted from single-dose data (Purkins et al., 2003). Genetic polymorphisms of *CYP2C19* are also a major determinant of the wide pharmacokinetic (PK) variability in voriconazole. Drug exposures from multiple doses of poor metabolizers (PMs) are almost 3 times higher than those from normal metabolizers (NMs) (Lee et al., 2012). Regarding age-related changes, the total body clearance in children is approximately 2–3-fold higher than that in adults (Levêque et al., 2006). In addition, oral bioavailability in children (45–66%) is only half of that in adults (96%) (Schulz et al., 2019), and this may suggest that primarily gut wall metabolism is also increased in children (Zane and Thakker, 2014). These PK discrepancies may be explained by developmental differences in organs, tissues, and enzymes (Kearns et al., 2003), resulting in an increased ratio of hepatic mass to total body mass and a higher clearance of CYP enzymes in children (Walsh et al., 2010).

The physiologically based pharmacokinetic (PBPK) model combines the knowledge of system-specific factors and drug-specific factors with mathematical modeling methods to quantitatively predict the PK characteristics of drug absorption, distribution, metabolism, and excretion (Maharaj and Edginton, 2014). Previously, an adult and pediatric PBPK model was established with hepatic *in vitro* data (Zane and Thakker, 2014). However, this model could not predict the nonlinear PKs of voriconazole and alterations in its metabolism over time. In another study, a whole-body PBPK model of voriconazole integrating the TDI of CYP3A4 and genetic polymorphisms of CYP2C19 was constructed (Li et al., 2020). It successfully captured the main PK characteristics of the drug in adults but overpredicted exposure to PMs after multiple doses.

Therefore, the objectives of the present study were to 1) establish an adult PBPK model of voriconazole, focusing on improving predictions of multiple-dose administration, especially in PMs; 2) extrapolate this model to children using age-related scaling methods; and 3) conduct simulations to facilitate the dose-optimization process. Due to the lack of data and high interpatient variability in the PK parameters observed following oral (p.o.) administration of voriconazole in children, the adult model was only extrapolated to intravenous (i.v.) administration in the pediatric model.

## Materials and Methods

### Workflow and Software

**FIGURE 1:**
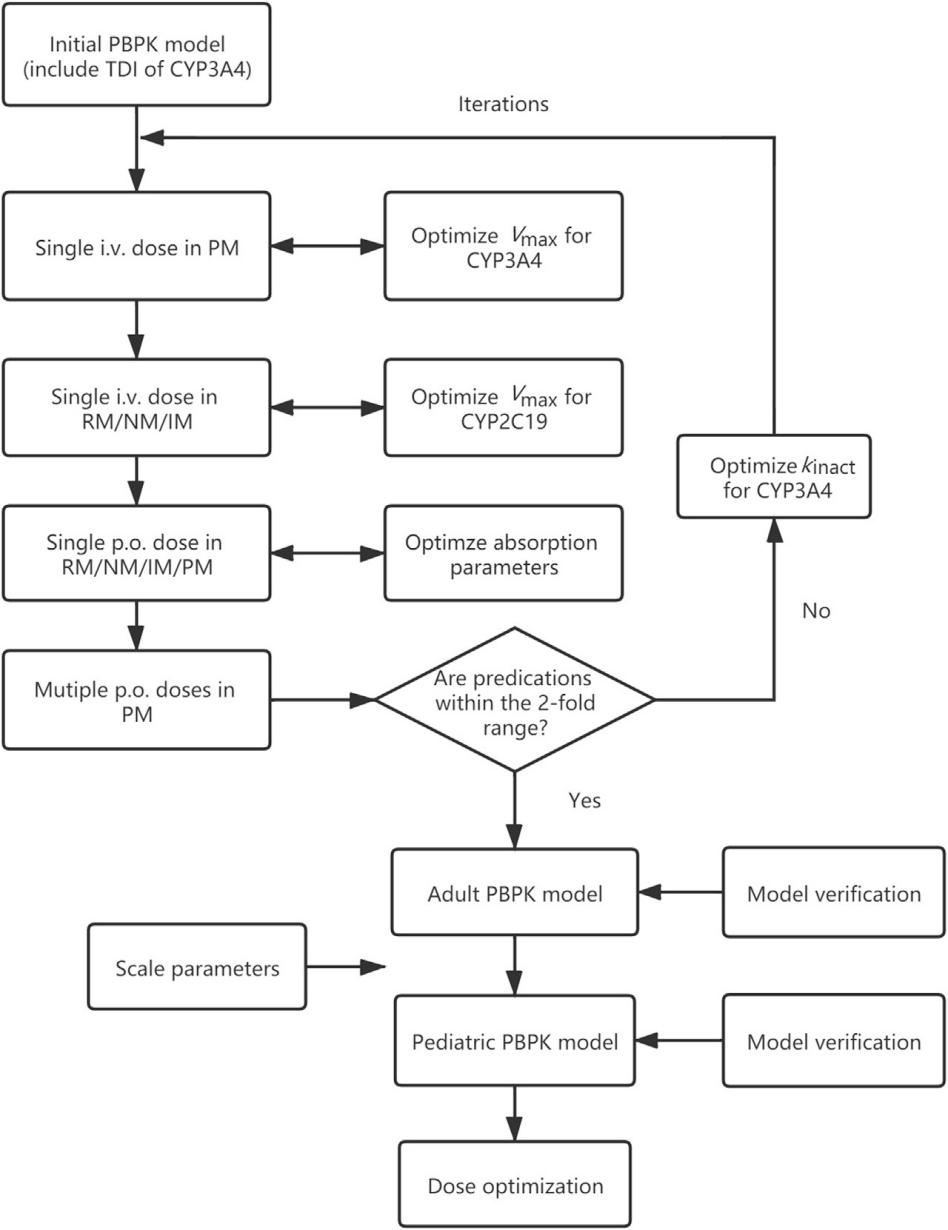
Adult and pediatric modeling workflow. PBPK model, physiologically based pharmacokinetic model; TDI, time-dependent inhibition; i.v., intravenously; p.o., orally; RM, rapid metabolizer; NM, normal metabolizer; IM, intermediate metabolizer; PM, poor metabolizer; *V*_max_, maximum velocity; *k*_inact_, maximum inactivation rate constant.

In this study, a PBPK model of voriconazole was developed and evaluated in the adult population, subsequently extrapolated to the pediatric population, and verified by comparing the simulated plasma exposure with the observed data. The final PBPK model was then used to simulate PK studies for pediatric dose optimization. The workflow of the model development is presented in Figure 1.

The modeling work was conducted in PK-Sim® (part of Open Systems Pharmacology (OSP) Suite version 8.0, www.open-systems-pharmacology.org). The published data were digitized by GetData Graph Digitizer version 2.26 (getdata-graph-digitizer.com).

### Adult Model Development

PK profiles following i.v. and p.o. administration were searched in PubMed, Embase and the Cochrane Library using the terms “voriconazole” and “pharmacokinetic”. The clinical trials with dosage, concentration, and *CYP2C19* genotype information in healthy adults were included.

A generic whole-body standard model for small molecules was selected in PK-Sim, and default system-dependent physiological parameters were implemented. With the assumption that CYP2C19, CYP3A4, and CYP2C9 are responsible for the metabolism of voriconazole, reverse transcription-polymerase chain reaction (RT-PCR) profiles were used to determine tissue expression distributions of these three enzymes, with hepatic reference concentrations of 0.76, 4.32, and 3.84 μmol/L, respectively, for CYP2C19 NMs (*1/*1). The abundance of ultrarapid metabolizers (UMs: *17/*17), rapid metabolizers (RMs: *1/*17), intermediate metabolizers (IMs: 1/*2, *1/*3, *2/*17), and PMs (2/*2, *2/*3, *3/*3) of CYP2C19 (Moriyama et al., 2018) was calculated using the scale factor estimated by Steere et al. (2015), and the values were 1.41, 1.36, 0.29(IMs with *1)/0.63(IMs with *17), and 0 μmol/L, respectively. If the genotype was not explicitly distinguished and mentioned, 0.46 was used for IMs. Due to the limited available data for UMs, the adult PBPK model was only built in CYP2C19 RMs, NMs, IMs and PMs.

Drug-specific parameters such as physicochemical properties and PK characteristics describing absorption, distribution, metabolism, and excretion were obtained from the literature, as shown in Table 1. Tissue-to-plasma partition coefficients were calculated using the Poulin and Theil method. Cellular permeabilities were predicted using the PK-Sim standard method. The enzymatic clearance process was quantified using Michaelis-Menten kinetics (Michaelis et al., 2011). The initial value of the Michaelis-Menten constant (*K*_m_) for CYP2C19, CYP3A4, and CYP2C9 was 3.5, 11, and 20 μmol/L, and the maximum velocity (*V*_max_) was set to 1.19, 2.3, and 0.0556 pmol/min^−1^/pmol, respectively (Hyland et al., 2003; Damle et al., 2011). As the ratio of AUC within the dosing interval after multiple doses to AUC from zero to infinity after a single dose (AUC_τ(multipole dose)_/AUC_inf(single dose)_) is larger than 2 (Purkins et al., 2003), the time-dependent inhibition of CYP3A4 was integrated into the model using Eq. 1.

$$\frac{dE_{\text{cat}}\left(t\right)}{dt}=k_{\deg}*E_{0}-\left(k_{\deg}+\frac{k_{\text{inact}}*I\left(t\right)}{K_{I}+I\left(t\right)}\right)*E_{\text{cat}}\left(t\right),$$ (1)

d*E*_cat_/dt describes the turnover of the enzyme, where *k*_deg_ is turnover rate constant, *E*_0_ is the initial enzyme concentration, I is the inhibitor concentration, *k*_inact_ is the maximum inactivation rate constant, and *K*_*I*_ is the inhibition concentration when reaching 50% of *k*_inact_. The initial values of *k*_inact_ and *K*_*I*_ were set to 0.04 min^−1^ and 9.33 μmol/L, respectively, according to the results of an *in vitro* inactivity assay (Li et al., 2020). Other parameters related to TDI were set as software default values. The glomerular filtration rate (GFR) fraction was fixed to 1, as there was no evidence for reabsorption and tubular secretion. Initial formulation-related parameters were also obtained from the literature, with the dissolution time when 50% of the substance dissolved (D_T,50_) set to 30 min and dissolution shape parameter for Weibull function (shape factor for tablet) set to 1.29 (Li et al., 2020).

**TABLE 1:** Summary of input parameters of voriconazole PBPK model.

Parameter	Unit	Value	Source
Molecular weight	g/mol	349.3	Pfizer Label
fu	%	42	Pfizer Label
log*P*		1.65	Drug bank
pKa		1.76 (base)	Damle et al. (2011)
Solubility (pH)	mg/mL	3.2 (1.0)	Pfizer Canada Inc.
Specific intestinal permeability	cm/s	2.81 × 10^−5^	Damle et al. (2011)
Partition coefficients		Poulin and Theil	Damle et al. (2011)
Cellular permeabilities		PK-Sim standard	Li et al. (2020)
CYP3A4 *K*_m_	μmol/L	11	Murayama et al. (2007)
CYP3A4 *k*_cat_	min^−1^	2.3	Optimized
CYP2C19 *K*_m_	μmol/L	3.5	Damle et al. (2011)
CYP2C19 *k*_cat_	min^−1^	1.19	Damle et al. (2011)
CYP2C9 *K*_m_	μmol/L	20	Hyland et al. (2003)
CYP2C9 *k*_cat_	min^−1^	0.0556	Hyland et al. (2003)
GFR fraction		1	
CYP3A4 *k*_inact_	min^−1^	0.04	Li et al. (2020)
CYP3A4 *K*_I_	μmol/L	9.33	Li et al. (2020)
D_T,50_ for tablet	min	30	Li et al. (2020)
Shape factor for tablet		1.29	Li et al. (2020)

fu, fraction of bound drug; logP, log of the partition coefficient between octanol and water; pKa, acid dissociation constant; K_m_, Michaelis-Menten constant; k_cat_, in vitro V_max_ per recombinant enzyme; GFR, glomerular filtration rate; k_inact_, maximum inactivation rate constant; K_I_, the inhibition concentration when reaching 50% of k_inact_; D_T,50_, the dissolution time when 50% of the substance dissolved; shape factor, the dissolution shape parameter for Weibull function.

The PBPK model was first established based on the initial values indicated above, and the *V*_max_ of CYP3A4 was optimized based on a single i.v. dose in CYP2C19 PMs, assuming that CYP3A4 contributes to almost 100% of the metabolism in this population. The propriety of CYP2C19-related parameters was then evaluated based on the observed PK parameters and plasma concentration profiles from a single i.v. dose in CYP2C19 RMs/NMs/IMs. In the next step, the specific intestinal permeability and formulation-related parameters, including D_T,50_ and shape factor for tablet, were inspected using data from studies following a single p.o. administration in RMs/NMs/IMs/PMs. If the fits were deemed inadequate, these three parameters were optimized based on p.o. data. Data from Scholz et al. (2009) was used for the above parameter optimization. The TDI-related parameter was optimized in the final step due to the lack of multiple i.v. clinical studies with genotype information. If the predicted PK parameters from multiple p.o. studies in PMs were within 2-fold of the observed values, the modeling process was complete. Otherwise, *k*_inact_ for CYP3A4 was optimized and the iterations of the above optimization steps were repeated.

### Adult Model Verification

The PK simulation of 100 virtual people for each clinical study was carried out, corresponding to the subject demographics (age range, proportion of male/female, and dosing regimens). The predictive performance was evaluated by visually comparing predicted concentration-time data with the observed data from the literature for initial verification. Ninety percent population prediction intervals covering the observed plasma concentration-time datasets were considered as a visual criterion for good performance. Next, the quantitative assessment was conducted by calculating the mean fold error (MFE) of PK parameters such as the area under the plasma concentration-time curve (AUC) and maximum concentrations (C_max_), expressed as the ratio of predicted to observed mean values. The model was acceptable if it met the 0.5- to 2.0-fold limit, and a more stringent criterion was the 0.8- to 1.25-fold range.

### Pediatric Model Development

PK profiles following i.v. administration were searched in PubMed, Embase and the Cochrane Library using the terms “voriconazole”, “pharmacokinetic”, and children-related items “infant”, “child”, “children”, “pediatric”, and “adolescent”. Studies with sufficient information for dosage, concentration, and CYP2C19 genotype were included.

Drug-specific parameters defined in the adult PK data were kept constant for the pediatric PBPK model.

Developmental changes in anatomical and physiological parameters such as weight, height, organ volumes, blood flows, organ composition, and plasma protein concentration in PK-Sim® are based on the population data from previous studies (NHANES III, 1997; ICRP, 2002). These algorithms were used to generate virtual pediatric populations. For the age-dependent hepatic clearance, default CYP2C19, CYP3A4 and CYP2C9 ontogeny information is described in the online PK-Sim Ontogeny Database Open Systems Pharmacology (2018). In summary, the activity of CYP2C19, CYP3A4 and CYP2C9 increases after birth and reaches the adult level over approximately 1, 4 and 1 year, respectively. The model using these default ontogeny profiles overpredicted the exposure in children; therefore, the ontogeny factors of CYP enzymes were estimated based on a recent meta-analysis using *in vivo* data (Upreti and Wahlstrom., 2016; Supplementary Figure S1). These data suggested that in childhood, CYP2C19, CYP3A4, and CYP2C9 exhibit maximal activities beyond the levels in adults.

### Pediatric Model Verification

The PBPK model performance in children was evaluated using the quantitative verification described in **Adult Model Verification**. As some pediatric clinical trials (Walsh et al., 2010) did not clearly show results according to the *CYP2C19* genotype as in adults, these data were verified by adding up different genotype results in the model based on genotype ratios from the literature. The visual check was not conducted due to the lack of available plasma concentration-time curves with gene information.

### Pediatric Dose Optimization

A 100-patients simulation was generated for each subpopulation classified by age (2-6 and 6-12 years) and CYP2C19 genotype (NMs, IMs and PMs). The individual AUC_24h_ (AUC during a 24-h period) was estimated.

The probability of reaching a ratio of free drug (unbound drug concentration) AUC_24h_ to minimum inhibitory concentration greater than or equal to 25 (*f*AUC_24h_/MIC ≥ 25) was considered to be the PK/pharmacodynamic (PD) index (Wang et al., 2015). The fraction of unbound drug was set to 42% (US FDA, 2005), with the assumption that this value is similar between children and adults (Yanni et al., 2008). Voriconazole MIC distributions for *Aspergillus* (4 species) and *Candida* (14 species) infections were obtained from the European Committee on Antimicrobial Susceptibility Testing database (2020); (Supplementary Figure S2). The probability of target attainment (PTA) at each MIC and the cumulative fraction of response (CFR) for the overall MIC distribution for each species were calculated. CFR values of all species in one genus larger than or equal to 80% was considered an appropriate dosage regimen (Mangal et al., 2018).

## Results

### Adult Model Verification

**FIGURE 2:**
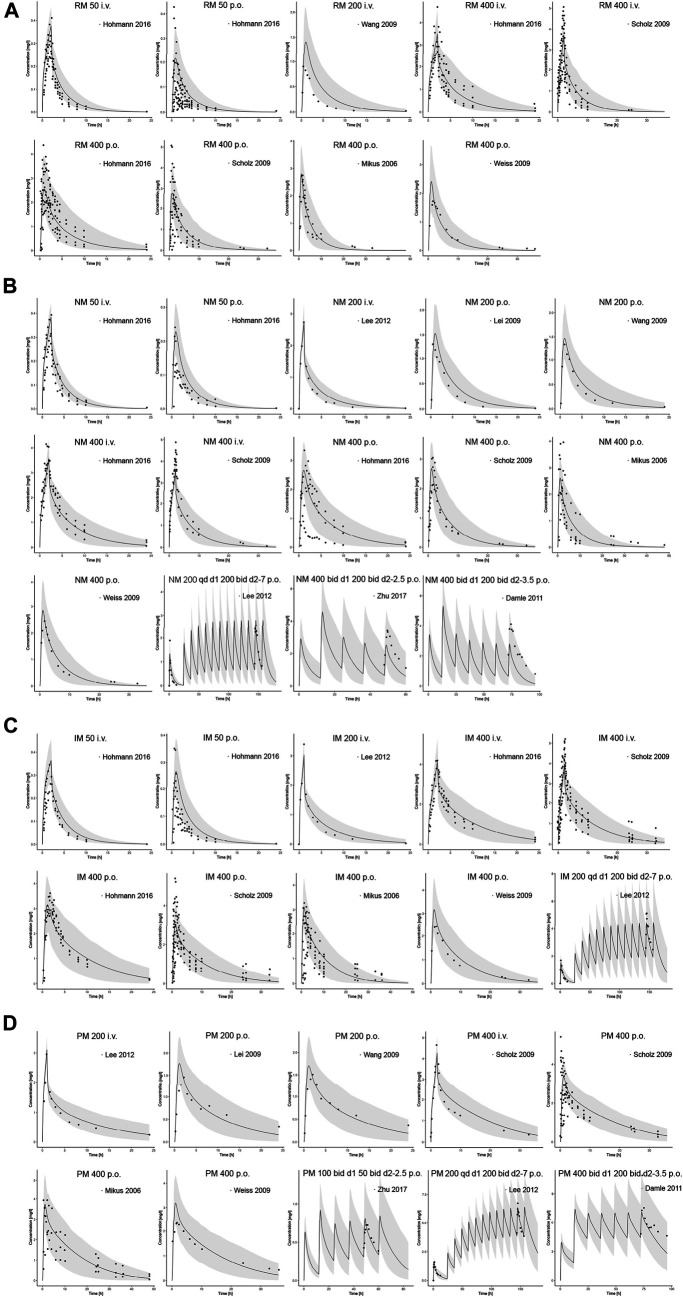
Comparison of adult PBPK model predicted plasma concentration-time profiles (lines) vs. clinical observed data (symbols) in **(A)** RM, **(B)** NM, **(C)** IM and **(D)** PM. PBPK predictions are presented as mean simulated concentrations (black line) with their 5th to 95th percentiles (grey area). i.v., intravenously; p.o., orally; RM, rapid metabolizer; NM, normal metabolizer; IM, intermediate metabolizer; PM, poor metabolizer.

**FIGURE 3:**
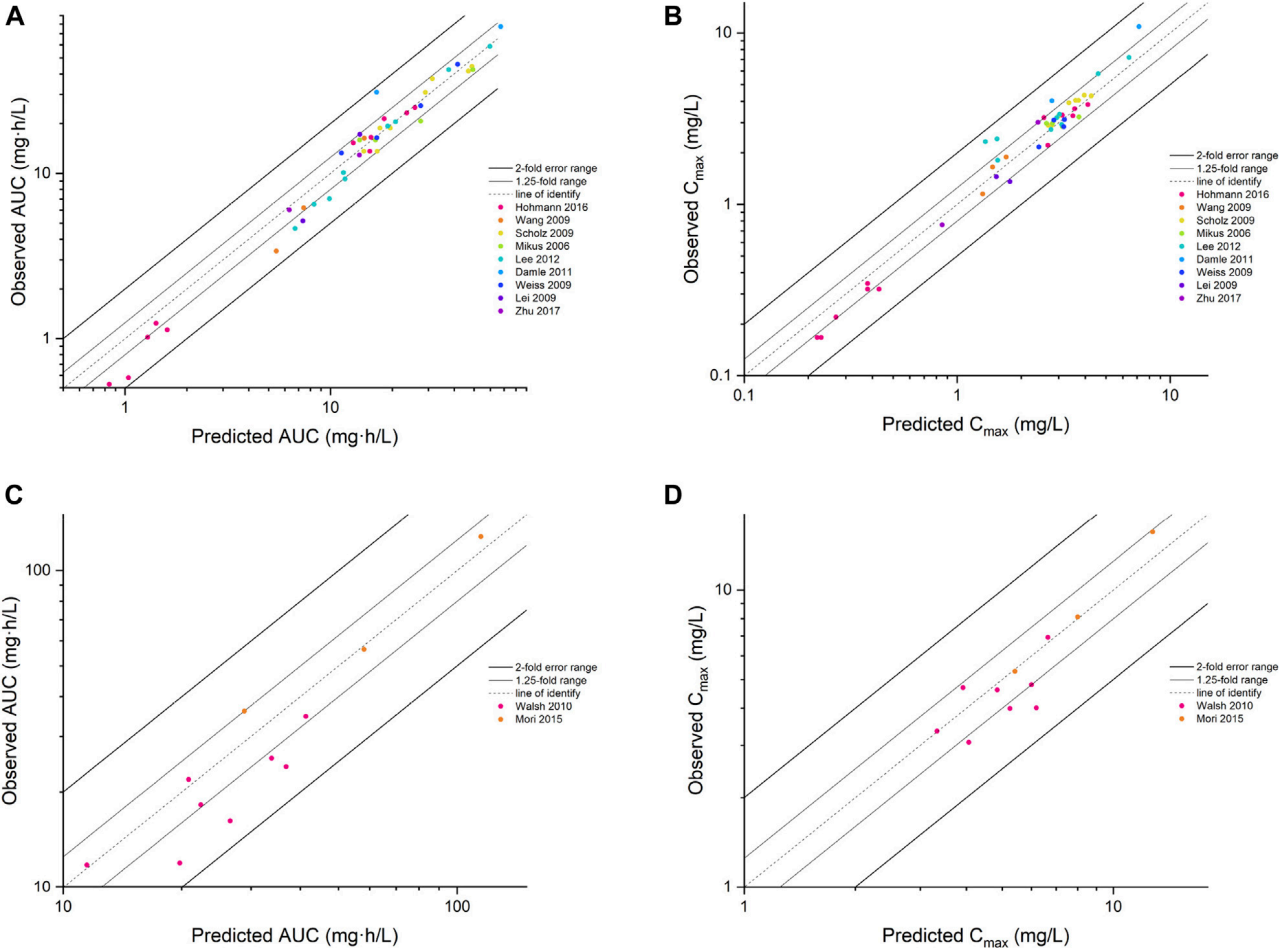
The goodness of fit plot for the PBPK model. Predicted vs. observed AUC for adults **(A)** and children **(C)**, C_max_ for adults **(B)** and children **(D)**. AUC, area under the concentration–time curve; C_max_, maximum concentration.

 Input parameters of voriconazole PBPK model were summarized in Table 1. A proper fit was achieved in the adult model, as shown in Figure 2, and most of the observed concentrations fell within the 5% and 95% concentration-time prediction intervals. All PK parameters were predicted to be within the 2-fold difference, with 78% of predicted/observed AUC ratios and 85% of C_max_ ratios within the 1.25-fold difference (Table 2; Figure 3). The prediction of PMs following multiple doses was satisfactory.

**TABLE 2:** Summary of voriconazole pharmacokinetic parameters in clinical studies of healthy adults and comparison with model predicted values.

*CYP2C19* genotype	Dose (mg)	Route	Male (%)	Age in years (age range group)	Pharmacokinetic parameters	Predicted	Observed	Pre/ Obs ratio	References
RM	50, sig	i.v. (120)	63	30 (24–53)	AUC_obs_	1.28	1.02	1.25	Hohmann et al. (2016)
					C_max_	0.38	0.320	1.19	
	50, sig	p.o. (tab)	63	30 (24–53)	AUC_obs_	0.76	0.40	1.9	Hohmann et al. (2016)
					C_max_	0.22	0.167	1.32	
	200, sig	p.o. (tab)	100	21 ± 2*	AUC_obs_	5.45	3.39	1.61	Wang et al. (2009)
					C_max_	1.32	1.15	1.15	
	400, sig	i.v. (120)	71	30 (24–53)	AUC_obs_	15.8	16.5	0.96	Hohmann et al. (2016)
					C_max_	3.50	3.29	1.06	
	400, sig	i.v. (120)	67	25 (23–28)	AUC_obs_	17.4	18.8	0.93	Scholz et al. (2009)
					C_max_	3.72	4.05	0.92	
	400, sig	p.o. (tab)	71	30 (24–53)	AUC_obs_	13.0	15.3	0.85	Hohmann et al. (2016)
					C_max_	2.56	3.21	0.90	
	400, sig	p.o. (tab)	67	25 (23–28)	AUC_obs_	14.6	13.6	1.07	Scholz et al. (2009)
					C_max_	2.79	2.90	0.96	
	400, sig	p.o. (cap)	0	29 (24–37)	AUC_obs_	13.9	15.9	0.87	Mikus et al. (2006)
					C_max_	2.81	2.97	0.95	
	400, sig	p.o. (cap)	100	27 (24–37)	AUC_inf_	11.3	13.3	0.85	Weiss et al. (2009)
					C_max_	2.42	2.16	1.12	
NM	50, sig	i.v. (120)	100	35 (24–46)	AUC_obs_	1.42	1.24	1.15	Hohmann et al. (2016)
					C_max_	0.38	0.345	1.10	
	50, sig	p.o. (tab)	100	35 (24–46)	AUC_obs_	0.84	0.53	1.58	Hohmann et al. (2016)
					C_max_	0.23	0.167	1.38	
	200, sig	i.v. (60)	100	26.7 ± 2.9*	AUC_inf_	8.33	6.51	1.28	Lee et al. (2012)
					C_max_	2.76	2.74	1.01	
	200, sig	p.o. (tab)	100	22 ± 1.5*	AUC_obs_	7.35	5.16	1.42	Lei et al. (2009)
					C_max_	1.53	1.45	1.06	
	200, sig	p.o. (tab)	100	21 ± 2	AUC_obs_	7.41	6.18	1.20	Wang et al. (2009)
					C_max_	1.47	1.65	0.89	
	200, qd, d1	p.o. (NA)	100	26.7 ± 2.9*	AUC_τ_	6.74	4.64	1.45	Lee et al. (2012)
					C_max_	1.36	2.32	0.59	
	200, bid, d2-7	p.o. (NA)	100	26.7 ± 2.9*	AUC_τ_	19.0	19.3	0.98	Lee et al. (2012)
					C_max_	2.94	3.21	0.92	
	200, bid, d2-2.5 (400, bid, d1)	p.o. (NA)	83	27 (18–45)	AUC_τ_	15.5	12.9	1.20	Zhu et al. (2017)
					C_max_	2.49	3.01	0.83	
	200, bid, d2-3.5 (400, bid, d1)	p.o. (NA)	100	29 (22–43)	AUC_12_	16.8	31.0	0.54	Damle et al. (2011)
					C_max_	2.79	4.02	0.69	
	400, sig	i.v. (120)	100	35 (24–46)	AUC_obs_	18.3	21.4	0.86	Hohmann et al. (2016)
					C_max_	3.57	3.61	0.99	
	400, sig	i.v. (120)	50	31 (24–38)	AUC_obs_	19.59	18.8	1.04	Scholz et al. (2009)
					C_max_	3.60	4.05	0.89	
	400, sig	p.o. (tab)	100	35 (24–46)	AUC_obs_	15.6	13.6	1.15	Hohmann et al. (2016)
					C_max_	2.67	2.21	1.21	
	400, sig	p.o. (tab)	50	31 (24–38)	AUC_obs_	16.9	13.6	1.24	Scholz et al. (2009)
					C_max_	2.68	2.90	0.92	
	400, sig	p.o. (cap)	100	28 (25–31)	AUC_obs_	16.6	15.9	1.04	Mikus et al. (2006)
					C_max_	2.63	2.97	0.89	
	400, sig	p.o. (cap)	100	27 (22–31)	AUC_inf_	16.8	16.4	1.02	Weiss et al. (2009)
					C_max_	2.85	3.10	0.92	
IM	50, sig	i.v. (120)	75	30 (25–34)	AUC_obs_	1.61	1.13	1.42	Hohmann et al. (2016)
					C_max_	0.43	0.32	1.34	
	50, sig	p.o. (tab)	75	30 (25–34)	AUC_obs_	1.04	0.58	1.79	Hohmann et al. (2016)
					C_max_	0.27	0.22	1.23	
	200, sig	i.v. (60)	100	24.7 ± 2.7*	AUC_inf_	11.6	10.1	1.15	Lee et al. (2012)
					C_max_	3.03	3.36	0.90	
	200, qd, d1	p.o. (NA)	100	24.7 ± 2.7*	AUC_τ_	9.90	7.02	1.41	Lee et al. (2012)
					C_max_	1.55	1.81	0.86	
	200, bid, d2-7	p.o. (NA)	100	24.7 ± 2.7*	AUC_τ_	37.7	42.4	0.89	Lee et al. (2012)
					C_max_	4.60	5.78	0.80	
	400, sig	i.v. (120)	63	26 (24–32)	AUC_obs_	31.3	37.4	0.84	Scholz et al. (2009)
					C_max_	3.96	4.33	0.91	
	400, sig	i.v. (120)	75	30 (25–34)	AUC_obs_	25.8	25.0	1.03	Hohmann et al. (2016)
					C_max_	4.11	3.82	1.08	
	400, sig	p.o. (tab)	75	30 (25–34)	AUC_obs_	23.5	23.2	1.01	Hohmann et al. (2016)
					C_max_	3.12	3.32	0.94	
	400, sig	p.o. (tab)	63	26 (24–32)	AUC_obs_	29.0	30.9	0.94	Scholz et al. (2009)
					C_max_	3.00	3.28	0.91	
	400,sig	p.o. (cap)	78	26 (22–33)	AUC_obs_	27.5	20.7	1.33	Mikus et al. (2006)
					C_max_	3.12	2.85	1.09	
	400,sig	p.o. (cap)	100	26 (22–33)	AUC_inf_	27.6	25.7	1.07	Weiss et al. (2009)
					C_max_	3.17	2.84	1.12	
PM	50, bid, d2-2.5 (100, bid, d1)	p.o. (NA)	100	29 (24–45)	AUC_τ_	6.32	6.00	1.05	Zhu et al. (2017)
					C_max_	0.85	0.76	1.12	
	200, sig	i.v. (60)	100	27.3 ± 3.6*	AUC_inf_	20.8	20.5	1.01	Lee et al. (2012)
					C_max_	3.10	2.92	1.06	
	200, sig	p.o. (tab)	100	21.6 ± 2.2*	AUC_obs_	13.9	17.2	0.81	Lei et al. (2009)
					C_max_	1.77	1.36	1.30	
	200, sig	p.o. (tab)	100	21 ± 2	AUC_obs_	14.6	16.3	0.90	Wang et al. (2009)
					C_max_	1.70	1.89	0.90	
	200, qd, d1	p.o. (NA)	100	27.3 ± 3.6*	AUC_τ_	11.8	9.25	1.28	Lee et al. (2012)
					C_max_	1.54	2.41	0.64	
	200, bid, d2-7	p.o. (NA)	100	27.3 ± 3.6*	AUC_τ_	59.8	58.7	1.02	Lee et al. (2012)
					C_max_	6.42	7.21	0.89	
	200, bid, d2-3.5 (400, bid, d1)	p.o. (NA)	100	29 (22–43)	AUC_12_	67.4	77.1	0.87	Damle et al. (2011)
					C_max_	7.14	10.9	0.66	
	400, sig	i.v. (120)	50	30 (20–37)	AUC_obs_	48.9	44.4	1.10	Scholz et al. (2009)
					C_max_	4.27	4.30	0.99	
	400, sig	p.o. (tab)	50	30 (20–37)	AUC_obs_	47.0	41.6	1.13	Scholz et al. (2009)
					C_max_	3.35	3.91	0.86	
	400, sig	p.o. (cap)	33	29 (19–37)	AUC_obs_	49.4	42.4	1.17	Mikus et al. (2006)
					C_max_	3.74	3.24	1.15	
	400, sig	p.o. (cap)	100	31 (19–37)	AUC_inf_	41.6	45.7	0.91	Weiss et al. (2009)
					C_max_	3.19	3.13	1.02	

d, day; sig, single dose; qd, once daily; bid, twice daily; i.v., intravenously; p.o., orally; tab, tablet; cap, capsule; Obs, observed value from clinical studies; Pre, predicted value from PBPK model; RM, rapid metabolizer; NM, normal metabolizer; IM, intermediate metabolizer; PM, poor metabolizer; AUC_obs_, area under the concentration-time curve from zero to the maximum observed time; AUC_inf_, area under the concentration-time curve from zero to infinity; AUC_τ_, area under the concentration-time curve within the dosing interval; AUC_12_, area under the concentration-time curve within 12 h; C_max_, maximum concentration; NA, not available; * mean ± (SD).

### Pediatric Model Verification

The PBPK model well captured the pharmacokinetic features in children after integrating *in vivo* ontogeny profiles. The PBPK simulation results were consistent with the observed plasma concentration-time profiles of different dosages after i.v. administration. All corresponding model-predicted concentrations fell within the 2-fold prediction error, with 73% of C_max_ values falling within the 1.25-fold prediction error (Table 3; Figure 3).

**TABLE 3:** Summary of voriconazole pharmacokinetic parameters in pediatric clinical studies and comparison with model predicted values.

*CYP2C19* genotype	Dose (mg/kg)	Route	Male (%)	Age in years (age range group)	Pharmacokinetic parameters	Predicted	Observed	Pre/obs ratio	References
58% NM + 42% IM	6 bid d1	i.v. (120)	75	3.7 (2–6)	AUC_τ_	11.49	11.77	0.98	Walsh et al. (2010)
	4 bid d2-d4	i.v. (80)			C_max_	3.33	3.35	0.99	
	6 bid d5-d8	i.v. (120)	75	3.7 (2–6)	AUC_τ_	20.82	21.93	0.95	
					C_max_	3.92	4.69	0.83	
73% NM + 27% IM	6 bid d1	i.v. (120)	75	8.7 (6–12)	AUC_τ_	19.78	11.95	1.66	
	4 bid d2-d4	i.v. (80)			C_max_	4.06	3.07	1.32	
	6 bid d5-d8	i.v. (120)	75	8.7 (6–12)	AUC_τ_	36.83	24.05	1.53	
					C_max_	6.19	4.01	1.54	
75% NM + 17% IM + 8% PM	6 bid d1-d4	i.v. (120)	45.8	2.8 (2–6)	AUC_τ_	22.39	18.22	1.23	
					C_max_	4.85	4.61	1.05	
	8 bid d5-d8	i.v. (160)	45.8	2.8 (2–6)	AUC_τ_	33.84	25.57	1.32	
					C_max_	6.00	4.80	1.25	
NM	6 bid d1-d4	i.v. (120)	45.8	8.1 (6–12)	AUC_τ_	26.57	16.23	1.64	
					C_max_	5.25	3.99	1.32	
	8 bid d5-d8	i.v. (160)	45.8	8.1 (6–12)	AUC_τ_	41.30	34.68	1.19	
					C_max_	6.65	6.92	0.96	
NM	9 bid d1	i.v. (180)	42.9	9.2 (3–14)	AUC_τ_	28.87	36.0	0.80	Mori et al. (2015)
	8 bid d2-d7	i.v. (160)			C_max_	5.42	5.32	1.02	
IM	9 bid d1	i.v. (180)	42.9	9.2 (3–14)	AUC_τ_	58.09	56.4	1.13	
	8 bid d2-d7	i.v. (160)			C_max_	8.01	8.12	0.99	
PM	9 bid d1	i.v. (180)	42.9	9.2 (3–14)	AUC_τ_	115	128	0.90	
	8 bid d2-d7	i.v. (160)			C_max_	12.78	15.70	0.81	

bid, twice daily; d, day; i.v., intravenously; Obs, observed value from clinical studies; Pre, predicted value from PBPK model; NM, normal metabolizer; IM, intermediate metabolizer; PM, poor metabolizer; AUC_τ_, area under the concentration-time curve within the dosing interval; C_max_, maximum concentration.

### Pediatric Dose Optimization

**FIGURE 4:**
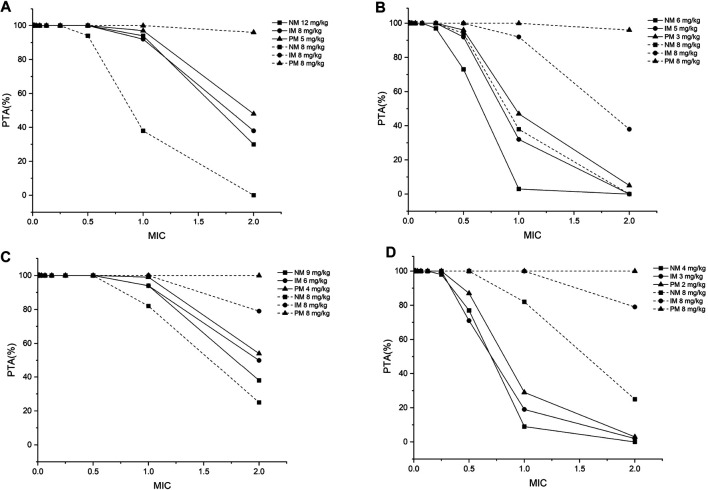
The minimum probability that targets pharmacokinetic/pharmacodynamic index of *f*AUC_24h_/MIC ≥ 25 among all species achieved at a specific MIC for each *CYP2C19* phenotype at the recommended dosage from the medication label (8 mg/kg) and recommended dose from the PBPK model for **(A)** children aged 2–6 years, infected with *Aspergillus* spp.; **(B)** children aged 2–6 years, infected with *Candida* spp.; **(C)** children aged 6–12 years, infected with *Aspergillus* spp.; **(D)** children aged 6–12 years, infected with *Candida* spp. PTA, probability of target attainment; MIC, minimum inhibitory concentration; NM, normal metabolizer; IM, intermediate metabolizer; PM, poor metabolizer; all the dosages are for the BID dosing regimens.

Twelve pediatric groups were constructed for dose design, and the results are shown in Table 4. IMs with *17 have a similar CYP2C19 abundance to NMs and the recommended dosage for NMs can be a reference for this subpopulation. Therefore, the following IM dosage recommendation is for IMs with *1. Figure 4 shows the contrast of the minimum PTA among different species in two fungal genera at each specific MIC between the following scenarios: one is administered the recommended maintenance dose from the current medication label (8 mg/kg, twice daily (BID)), and the other one is administered the recommended dose determined from the PBPK model. The results suggested that, for the BID dosing regimens, intravenous doses should be adjusted to 12 mg/kg for NMs, 8 mg/kg for IMs, and 5 mg/kg for PMs for 2–6-year-old children infected with *Aspergillus* spp. As children grow older, the recommended dose decreases, specifically 9 mg/kg for NMs, 6 mg/kg for IMs, and 4 mg/kg for PMs infected with *Aspergillus* spp. When the infectious fungal genus is *Candida* spp., approximately half of the above dosages are recommended to attain the appropriate drug exposure using *f*AUC_24h_/MIC as the indicator, due to notable differences in these two fungi in terms of susceptibility to voriconazole.

**TABLE 4:** Recommended dosages and CFR values with a target value of *f*AUC_24h_/MIC ≥ 25.

Population	Infectious fungal genera			
	*Aspergillus* spp.		*Candida* spp.	
	Dosage mg/kg	CFR %	Dosage mg/kg	CFR%
Children aged 2–6 years				
NM	12	80.1	6	80.2
IM	8	80.9	5	90.0
PM	5	86.0	3	91.9
Children aged 6–12 years				
NM	9	82.0	4	82.4
IM	6	84.9	3	82.0
PM	4	88.6	2	88.4

fAUC2_24h_/MIC ≥ 25, ratio of free drug AUC during a 24-h period to minimum inhibitory concentration greater than or equal to 25; AUC, area under the concentration-time curve; NM, normal metabolizer; IM, intermediate metabolizer; PM, poor metabolizer; CFR, cumulative fraction of response; all the recommended dosages are for the BID dosing regimens.

## Discussion

The adult and pediatric PBPK model of voriconazole, which incorporated the TDI of CYP3A4, gene polymorphisms of *CYP2C19*, and developmental changes in physiology and metabolic enzymes, were able to describe the PKs in both populations. Subsequent simulations revealed that age, *CYP2C19* genotype, and infectious fungal genera influence target PK/PD index attainment and should be considered in dose optimization.

Voriconazole is often for long-term use, from weeks to months, in the prophylaxis and treatment of IFI (Benitez and Carver, 2019). Therefore, it is essential to elucidate the time-dependent PK characteristics of this medication following multiple doses. To accomplish this goal, the strategy used for model building in this study is slightly different from that used in other studies. TDI-related parameters have always been optimized in the final step in multiple studies. However, the resulting change in these values can affect the previous predictions of single-dose administration. Generally, retrograde clearance can be implemented in the model first, and then sensitivity analysis is conducted to determine the optimal value of uncertain parameters (Wu et al., 2014). However, due to the possible inaccuracies of data digitized from the literature, which may influence the sensitivity analysis, this method was not used. Instead, a “bottom-up” and “top-down” combination strategy incorporating manual iterations was used to facilitate the model building process. The adult PBPK model results showed satisfactory predictive performance for multiple-dose administration in all *CYP2C19* genotype populations.

There was an overprediction of exposure in children when default enzyme ontogeny profiles from *in vitro* experiments were utilized to extrapolate the adult model. Therefore, ontogeny factors based on a meta-analysis of *in vivo* CYP activity with age-related changes (Upreti and Wahlstrom, 2016) were implemented. In this meta-analysis, the maximal activity of CYP2C19, CYP3A4, and CYP2C9 in children exceeded the corresponding adult values. The higher capacity or expression of CYP enzymes in children was also corroborated from a previous *in vitro* experiment using human liver microsomes (Yanni et al., 2010). That experiment showed that the apparent *V*_max_ for voriconazole conversion to the N-oxide metabolite was approximately 3-fold higher in children than in adults. After incorporating the new ontogeny profiles, the predictive performance of the PBPK model was improved. Moreover, the clearance was approximately 2–3-fold higher in children than in adults in the simulation, which is consistent with a previous study (Walsh et al., 2004).

The final extrapolated model presented the PK features of voriconazole in the pediatric population following i.v. administration, except that the exposure of several 6–12-year-old children in the study by Walsh et al. was slightly overpredicted. There may be several reasons. First, the predicted PK parameters were calculated from a cohort of children with a certain percentage of different CYP2C19 metabolic types described in the baseline demographic data. However, a few children discontinued treatment due to adverse events. The metabolic types of these children were not disclosed, which may result in differences between the predicted and observed values. Furthermore, although children who were receiving drugs known to interact strongly with voriconazole, such as terfenadine and pimozide, were excluded, concomitant medications that could potentially interact with voriconazole were permitted, which might have resulted in altered PKs that were not considered in the PBPK model.

The results of dose optimization seem to be reasonable. Most of the recommended dosages for NM and IM children aged 2–12 years infected with *Aspergillus* spp*.* are similar to the dosages adopted by the European Medicines Agency (EMA) for children aged 2–11 years, specifically a loading i.v. dose of 9 mg/kg BID on day 1, followed by a maintenance dose of 8 mg/kg BID (European Medicines Association, 2012). They are also close to the loading dose of 7 mg/kg BID recommended by the Infectious Disease Society of America (IDSA) (Pappas et al., 2009), except for 12 mg/kg, which is the recommended dosage for 2–6-year-old NMs. Although the tolerance of higher dosage up to 10 mg/kg in children has been confirmed (Sano et al., 2016), further validation of this regimen's exposure and safety is needed before implementing it in clinical practice. In other subpopulations, dose adjustment is proposed based on several factors. First, younger children may have a relatively higher enzyme expression or activity (Upreti and Wahlstrom., 2016), contributing to higher metabolism and lower concentration. Thus, higher recommended doses in younger children, especially <5 years old, seem warranted (Soler-Palacín et al., 2011). Second, as CYP2C19 is the major enzyme in the metabolism of voriconazole (Barbarino et al., 2018), lower doses should be administered to people with alleles associated with enzymatic loss-of-function (Moriyama et al., 2017). Third, invasive fungal infection classification should be considered a key factor for antifungal therapy (Wang et al., 2016). The susceptibility of *Candida* spp. to voriconazole is higher than that of *Aspergillus* spp.; therefore, a reduced dose of voriconazole should be sufficient for infections involving the former (Perez-Pitarch et al., 2019).

The recommended dosage in this study should be deemed a reference for the initial dosing regimen because the target CFR value represented the overall distribution of MIC in the population and the clinical data utilized for model validation were mean drug exposure. It cannot completely solve the problem of high PK variability and replace the importance of therapeutic drug monitoring (Gastine et al., 2017). Subsequent dose adjustments should be conducted based on the individual MIC, drug concentration and clinical response (Muto et al., 2015).

One limitation of this study is that the model was established based on some fundamental assumptions. For example, CYP2C19, CYP3A4, and CYP2C9 account for the entire metabolism of voriconazole, and the metabolic pathway is the same in both adults and children. A study of human liver microsomes provided evidence that flavin-containing monooxygenase 3 (FMO3) contributes to higher clearance in children than in adults (Yanni et al., 2010). However, this was not integrated into our model due to the small effect on the main metabolite of voriconazole observed in previous recombinant FMO3 enzyme experiments (Yanni et al., 2008). Moreover, the limited available data on RMs made it impossible to recommend dosage for this subpopulation in children. The established model and the recommended dosage for other subpopulations may also require further verification in carefully designed clinical trials.

In conclusion, the developed PBPK model of voriconazole provides a more accurate description of PK characteristics in adults following single and multiple i.v. and p.o. administrations, especially in PMs. It also predicts exposure in children following i.v. administration with good accuracy. Age, *CYP2C19* genotype, and infectious fungal genera were found to significantly influence the attainment of the PK/PD target in the simulation and thus should be considered for clinical dose selection.

## Data Availability

The original contributions presented in the study are included in the article/Supplementary Material, further inquiries can be directed to the corresponding author.
