# Supplementary material for: Application of a Physiologically Based Pharmacokinetic Model to Characterize Time-dependent Metabolism of Voriconazole in Children and Support Dose Optimization
Source: Front Pharmacol. 2021 Mar 17;12:636097. doi: 10.3389/fphar.2021.636097 (PMC8010309; doi:10.3389/fphar.2021.636097)
Supplement: Supplementary file 1 [file datasheet1.docx]

Supplementary Material


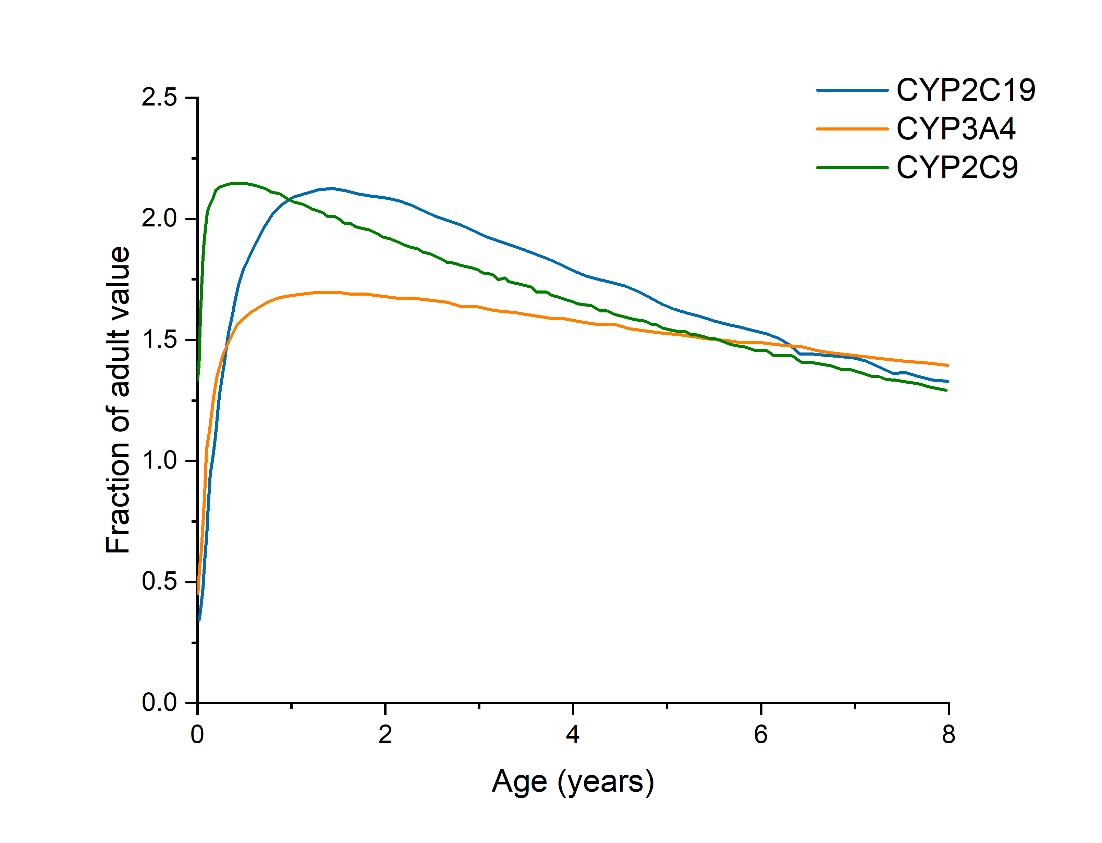


**Figure S1**. *In vivo* CYP ontogeny: CYP2C19 (blue), CYP3A4 (orange), CYP2C9(green) (Upreti and Wahlstrom., 2016).


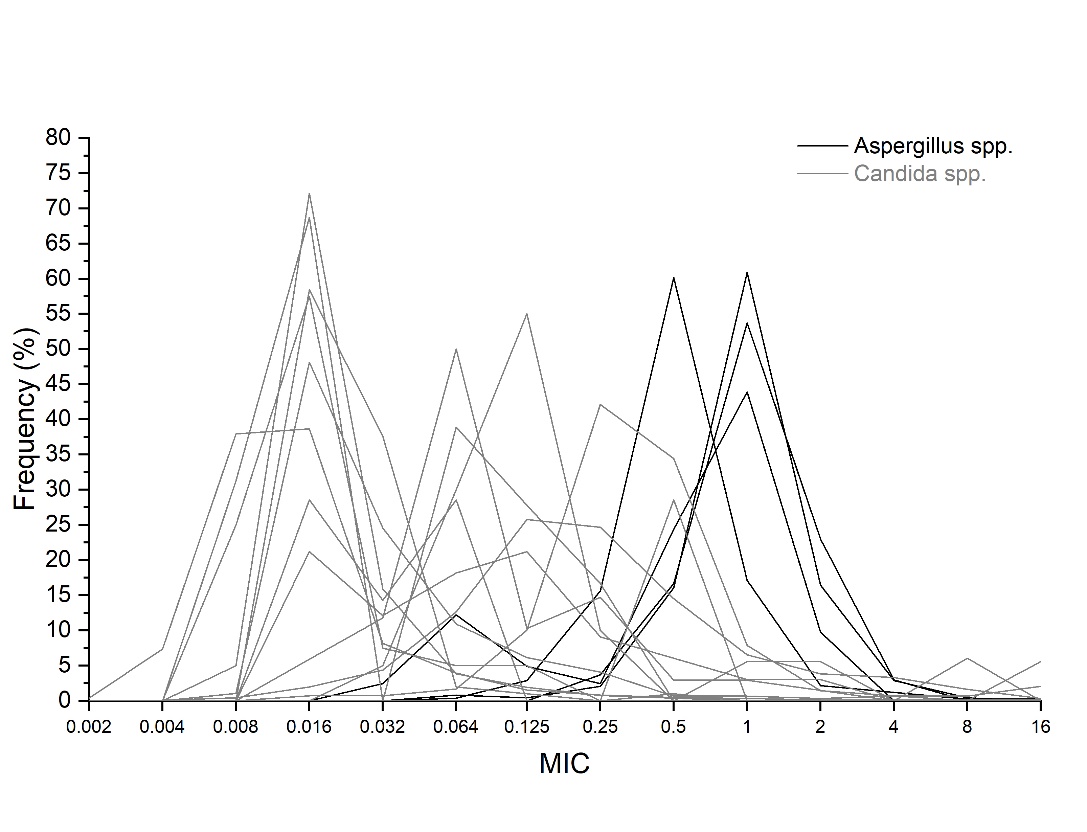


**Figure S2**. MIC distributions for *Aspergillus* spp. (4 species) and *Candida* spp. (14 species) from EUCAST database (2020). MIC, minimum inhibitory concentration; EUCAST, the European Committee on Antimicrobial Susceptibility Testing.

**References**

Upreti, V. V., and Wahlstrom, J. L. (2016). Meta-analysis of hepatic cytochrome P450 ontogeny to underwrite the prediction of pediatric pharmacokinetics using physiologically based pharmacokinetic modeling. J. Clin. Pharmacol. 56(3), 266–283. doi:10.1002/jcph.585

European Committee on Antimicrobial Susceptibility Testing. Available from: https://mic.eucast.org/Eucast2/SearchController/search.jsp?action=performSearch&BeginIndex=0&Micdif=mic&NumberIndex=50&Antib=152&Specium=-1 (Accessed Oct 27, 2020)
